# Detection of copeptin in peripheral blood of patients with aneurysmal subarachnoid hemorrhage

**DOI:** 10.1186/cc10575

**Published:** 2011-11-29

**Authors:** Xiang-Dong Zhu, Jing-Sen Chen, Feng Zhou, Qi-Chang Liu, Gao Chen, Jian-Min Zhang

**Affiliations:** 1 Department of Neurosurgery, The Second Affiliated Hospital, School of Medicine, Zhejiang University, 88 Jiefang Road, Hangzhou 310000, PR China

## Abstract

**Introduction:**

Copeptin has been proposed as a prognostic marker in acute illness. This study investigated the ability of copeptin to predict the disease outcome and cerebrovasospasm in the patients with aneurysmal subarachnoid hemorrhage.

**Methods:**

In this retrospective study, 303 consecutive patients were included. Upon admission, plasma copeptin levels were measured by enzyme-linked immunosorbent assay. The end points were mortality after 1 year, in-hospital mortality, cerebrovasospasm and poor functional outcome (Glasgow Outcome Scale score of 1-3) after 1 year.

**Results:**

Upon admission, plasma copeptin level in patients was statistically significantly higher than that in healthy controls. A multivariate analysis showed that plasma copeptin level was an independent predictor of poor functional outcome and mortality after 1 year, in-hospital mortality and cerebrovasospasm. A receiver operating characteristic curve showed that plasma copeptin level on admission predicted poor functional outcome and mortality after 1 year, in-hospital mortality and cerebrovasospasm of patients statistically significantly. The area under curve of the copeptin concentration was similar to those of World Federation of Neurological Surgeons (WFNS) score and modified Fisher score for the prediction of poor functional outcome and mortality after 1 year, and in-hospital mortality, but not for the prediction of cerebrovasospasm. In a combined logistic-regression model, copeptin improved the area under curve of WFNS score and modified Fisher score for the prediction of poor functional outcome after 1 year, but not for the prediction of mortality after 1 year, in-hospital mortality, and cerebrovasospasm.

**Conclusions:**

Copeptin level is a useful, complementary tool to predict functional outcome and mortality after aneurysmal subarachnoid hemorrhage.

## Introduction

Copeptin, the C-terminal part of the arginine vasopressin precursor peptide, is associated with the severity and outcome of critical illness, and therefore, has been proposed as a prognostic marker in acute illness [1-11]. Recently, it has been reported that plasma copeptin levels were also elevated in the patients with traumatic brain injury [11,12] and intracerebral hemorrhage [10,13] and ischemic stroke [8,9]; in these groups of patients, high copeptin levels were highly predictive for poor outcome. However, No published information exists to date about the association of copeptin with disease outcome and cerebrovasospasm after aneurysmal subarachnoid hemorrhage (SAH). The present study aimed to investigate the ability of copeptin to predict the disease outcome and cerebrovasospasm in the patients with aneurysmal SAH.

## Materials and methods

### Study population

Between July 2008 and March 2010, all patients with aneurysmal SAH confirmed by computerized tomography (CT) angiography with or without digital subtraction angiography who were admitted to Department of Neurosurgery, Second Affiliated Hospital, School of Medicine, Zhejiang University were evaluated in the study. Inclusion criteria were clinical history of SAH within the last 24 hrs before admission and the treatment by surgery or coiling within the 48 hrs after admission. Exclusion criteria were less than 18 years of age, existing previous head trauma, neurological diseases including ischemic or hemorrhagic stroke, use of antiplatelet or anticoagulant medication, and presence of other prior systemic diseases including uremia, liver cirrhosis, malignancy, chronic heart or lung disease, diabetes mellitus and hypertension.

A control group consisted of 150 healthy sex and age-matched subjects with normal results on brain magnetic resonance imaging and without vascular risk factors.

Written informed consent to participate in the study was obtained from the subjects or their relatives. This protocol was approved by the Ethics Committee of The Second Affiliated Hospital, School of Medicine, Zhejiang University before implementation.

### Clinical and radiological assessment

On arrival to the emergency department, a detailed history of vascular risk factors, concomitant medication, Glasgow Coma Scale (GCS) score, body temperature, heart rate, respiratory rate and blood pressure were taken. At admission, clinical severity was assessed using the World Federation of Neurological Surgeons (WFNS) score [14]. The initial CT was classified according to the modified Fisher score [15]. All CT scans were performed according to the neuroradiology department protocol. Investigators who read them were blinded to clinical information.

### Patient management

The type of treatment (surgery or coiling) was decided according to both location and size of the aneurysm by the neurosurgeon and the neuroradiologist. All patients received intravenous Nimodipine at a dose of 2 mg/h from admission until at least day 14, except during periods of uncontrolled increased intracranial pressure during which intravenous Nimodipine was discontinued. Seizures were systematically prevented by Sodium Valproate (200 mg × 3, per os). After surgery or coiling, those patients who had delayed ischemic neurological deficit or cerebrovasospasm were managed with 'triple H' therapy (hypertension with a mean arterial pressure goal greater than 100 mm Hg, hypervolemia and hemodilution with a goal hematocrit of 30) through 12 days after hemorrhage. An external ventricular drain was inserted in case of hydrocephalus on CT and in patients with a high WFNS grade (WFNS score of 3-5). Increased intracranial pressure was treated by cerebrospinal fluid drainage, mechanical ventilation, reinforcement of sedation, and, rarely, moderate hypothermia. CT was performed whenever clinical deterioration occurred to search for secondary complications such as hydrocephalus or ischemia.

Clinical onset of cerebral vasospasm was defined as the acute onset of a focal neurologic deficit or a change in the GCS score of 2 or more points. All suspected cases of cerebral vasospasms were confirmed by CT angiography and were then taken to the interventional radiology suite for cerebral angiography. Each vasospasm episode was treated with intra-arterial administration of Nimodipine as recently described. This therapy was repeated if necessary. Balloon Angioplasty was used as a second-line therapy when Nimodipine was judged insufficient. Computed tomography ischemia was referred to as delayed ischemia attributed to vasospasm.

### Determination of copeptin in plasma

The informed consents were obtained from study population or family members in all cases before the blood were collected. In the control group, venous blood was drawn at study entry. In the SAH patients, venous blood was drawn on admission. The blood samples were immediately placed into sterile EDTA test tubes and centrifuged at 1500 g for 20 minutes at 4°C to collect plasma. Plasma was stored at -70°C until assayed. The concentration of copeptin in plasma was analyzed by enzyme-linked immunosorbent assay (ELISA) using commercial kits (Cusabio biotech co. ltd, Wuhan, Hubei Province, China) in accordance with the manufactures' instructions. Intra-assay and inter-assay coefficients of variation were 4.2% and 6.8%. The blood samples were run in duplicate. Researchers running ELISAs were blinded to all patient details.

### End point

Participants were followed up until death or completion of one year after SAH. Their primary outcome was death (at 1 year or in-hospital) and their secondary outcomes were vasospasm and functional outcome at 1 year. The functional outcome was defined by Glasgow outcome scale (GOS) score. GOS was defined as follows: 1 = death; 2 = persistent vegetative state; 3 = severe disability; 4 = moderate disability; and 5 = good recovery [16]. GOS Scores were dichotomized in good and poor functional outcomes (GOS of 4-5 vs. GOS of 1-3). For follow-up, we used structure telephone interviews performed by 1 doctor, blinded to clinical information and copeptin levels.

### Statistical analysis

Statistical analysis was performed with SPSS 10.0 (SPSS Inc., Chicago, IL, USA) and MedCalc 9.6.4.0. (MedCalc Software, Mariakerke, Belgium). The normality of data distribution was assessed by the Kolmogorovor-Smirnov test or Shapiro-Wilk test. All values are expressed as mean ± standard deviation or counts (percentage) unless otherwise specified. Comparisons were made by using (1) chi-square test or Fisher exact test for categorical data, (2) unpaired Student *t *test for continuous normally distributed variables, and (3) the Mann-Whitney U-test for continuous non-normally distributed variables. The relations of copeptin to the poor functional outcome (GOS 1-3), death and cerebrovasospasm were assessed in a binary logistic-regression model. For multivariate analysis, we included the significantly different outcome predictors as assessed in univariate analysis. A receiver operating characteristic curve was configured to establish the cutoff point of plasma copeptin with the optimal sensitivity and specificity for predicting the poor functional outcome (GOS 1-3), death and cerebrovasospasm. In a combined logistic-regression model, we estimated the additive benefit of copeptin to other predictors (WFNS grade and Fisher grade). A *P *value of less than 0.05 was considered statistically significant.

## Results

### Study population characteristics

**Figure 1:**
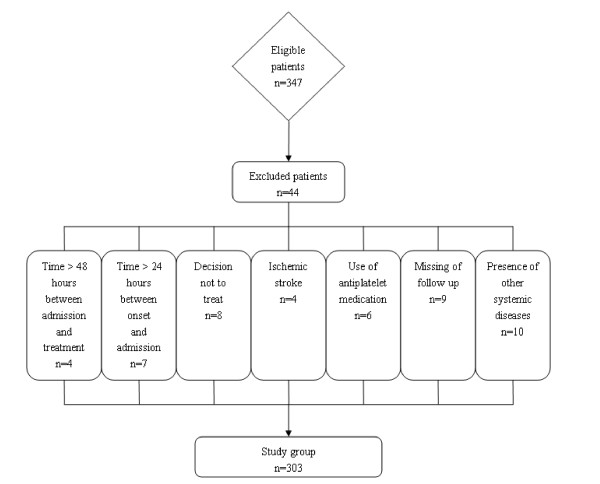
**Graph documenting patients' entry into the study from screening**.

During the recruitment period, 347 patients were admitted with an initial diagnosis of aneurysmal SAH, 312 (89.9%) patients fulfilled the inclusion criteria, and adequate data on admission and follow-up were available for 303 individuals (87.3%) who were finally included in the analysis (Figure 1). Table 1 summarized the demographic, clinical, laboratory and radiological data of the patients.

**Table 1:** The characteristics for 303 patients

Characteristics	
Sex (male/female)	131/172
Age (y)	43.9 ± 12.4
World Federation of Neurological Surgeons score on admission	2.3 ± 1.2
Modified Fisher score on admission	2.7 ± 1.0
Aneurysmal location	
Posterior communication artery	83 (27.4%)
Internal carotid artery	43 (14.2%)
Anterior communication artery	66 (21.8%)
Middle cerebral artery	45(14.9%)
Anterior cerebral artery	35 (11.6%)
Posterior cerebral artery	23 (7.6%)
Vertebral artery	8 (2.6%)
Surgery	186 (61.4%)
Aneurysmal size (mm)	7.2 ± 4.9
Rebleeding	16 (5.3%)
Acute hydrocephalus	90 (29.7%)
Intracerebral hemorrhage	39 (12.9%)
Intraventricular hemorrhage	72 (23.8%)
External ventricular drain	109 (36.0%)
Angiographic vasospasm	131 (43.2%)
Computed tomography ischemia	50 (16.5%)
Admission time (hr)	4.7 ± 3.6
Plasma-sampling time (hr)	6.7 ± 4.4
Seizure	44 (14.5%)
Plasma C-reactive protein level (mg/L)	7.1 ± 2.7
plasma D-dimer level (mg/L)	2.1 ± 0.9
Plasma copeptin level (pmol/L)	21.2 ± 9.0

Numerical variables were presented as mean ± standard deviation. Categorical variables were expressed as counts (percentage).

### The change in plasma copeptin level on admission in patients with SAH

**Figure 2:**
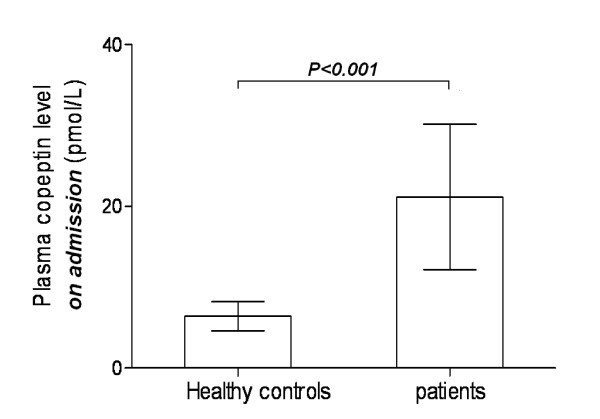
**Graph showing the change of plasma copeptin concentration in the patients with aneurysmal subarachnoid hemorrhage**. Data are expressed as mean ± standard deviation.

After SAH, plasma copeptin level on admission in patients was statistically significantly higher than that in healthy controls (21.2 ± 9.0 pmol/L vs. 6.4 ± 1.8 pmol/L; *P *< 0.001)(Figure 2).

### One-year mortality prediction

Forty-two patients (13.9%) died from SAH in one year. Higher plasma copeptin level was associated with one-year mortality, as well as other variables shown in the Table 2. When the above variables found to be significant in the univariate analysis were introduced into the logistic model, A multivariate analysis selected WFNS score, modified Fisher score and plasma copeptin level as the independent predictors for one-year mortality of patients (Table 3).

**Table 2:** The factors associated with one-year mortality

	Non-survivals (*n *= 42)	Survival (*n *= 261)	*P *value
Sex (male/female)	18/24	113/148	0.958
Age (y)	45.4 ± 13.4	43.6 ± 12.2	0.389
WFNS score on admission	4.0 ± 0.7	2.1 ± 1.0	<0.001
Modified Fisher score on admission	4.2 ± 0.6	2.5 ± 0.8	<0.001
Aneurysmal location			0.614
Posterior communication artery	8 (19.0%)	75 (28.7%)	
Internal carotid artery	6 (14.3%)	37(14.2%)	
Anterior communication artery	9 (21.4%)	57 (21.8%)	
Middle cerebral artery	7 (16.7%)	38 (14.6%)	
Anterior cerebral artery	6 (14.3%)	29 (11.1%)	
Posterior cerebral artery	4 (9.5%)	19 (7.3%)	
Vertebral artery	2 (4.8%)	6 (2.3%)	
Surgery	21 (50.0%)	165 (63.2%)	0.102
Aneurysmal size (mm)	11.1 ± 5.3	6.6 ± 4.5	<0.001
Rebleeding	10 (23.8%)	6 (2.3%)	<0.001
Acute hydrocephalus	25 (59.5%)	65 (24.9%)	0.001
Intracerebral hemorrhage	19 (45.2%)	20 (7.7%)	<0.001
Intraventricular hemorrhage	37 (88.1%)	35 (13.4%)	<0.001
External ventricular drain	38 (90.5%)	71 (27.2%)	<0.001
Angiographic vasospasm	38 (90.5%)	93 (35.6%)	<0.001
Computed tomography ischemia	18 (42.9%)	32 (12.3%)	<0.001
Admission time (hr)	5.5 ± 4.4	4.6 ± 3.5	0.128
Seizure	9 (21.4%)	35 (13.4%)	0.171
Plasma C-reactive protein level (mg/L)	8.7 ± 3.3	6.9 ± 2.6	<0.001
plasma D-dimer level (mg/L)	2.4 ± 1.0	2.0 ± 1.0	0.015
Plasma copeptin level (pmol/L)	31.4 ± 8.3	19.5 ± 8.0	<0.001

Numerical variables were presented as mean ± standard deviation. Categorical variables were expressed as counts (percentage). Numerical variables were analyzed by Mann-Whitney U-test or unpaired Student *t *test. Categorical variables were analyzed by chi-square test or Fisher exact test. n indicates number of patients; WFNS, World Federation of Neurological Surgeons.

**Table 3:** Multivariate analysis of factors predicting the one-year mortality among 303 patients

	Odds ratio	95% confidence interval	*P *value
WFNS score on admission	7.530	1.351~20.642	0.002
Modified Fisher score on admission	9.181	2.236~22.297	0.006
Aneurysmal size (mm)	1.182	0.914~1.324	0.361
Rebleeding	4.243	0.624~26.748	0.311
Acute hydrocephalus	1.249	0.230~25.387	0.401
Intracerebral hemorrhage	1.235	0.642~6.891	0.183
Intraventricular hemorrhage	3.822	0.893~11.402	0.064
External ventricular drain	1.105	0.611~4.008	0.397
Angiographic vasospasm	2.164	0.912~6.217	0.132
Computed tomography ischemia	1.472	0.881~5.438	0.271
Plasma C-reactive protein level (mg/L)	1.104	0.861~1.945	0.401
plasma D-dimer level (mg/L)	0.941	0.573~1.908	0.781
Plasma copeptin level (pmol/L)	2.307	1.324~6.974	0.004

The relation of copeptin to the mortality was assessed in a logistic-regression model. WFNS indicates World Federation of Neurological Surgeons.

**Figure 3:**
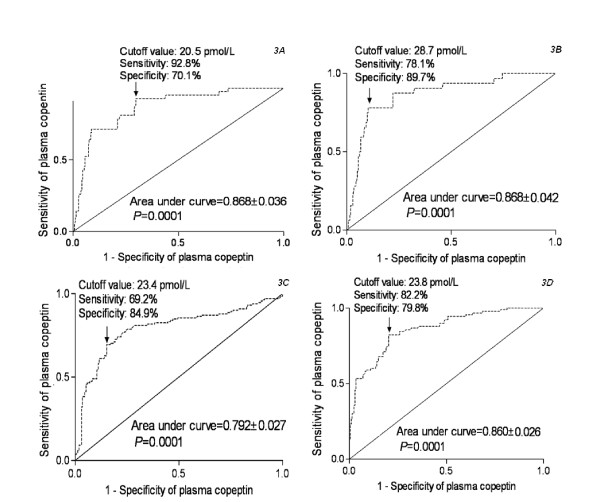
**Graph showing receiver operating characteristic curve analysis of plasma copeptin level for one-year mortality (3A), in-hospital mortality (3B), cerebrovasospasm (3C) and one-year poor functional outcome (3D)**.

A receiver operating characteristic curve showed that plasma copeptin level on admission predicted one-year mortality of patients statistically significantly (Figure 3A). The predictive value of the copeptin concentration was similar to those of WFNS score and modified Fisher score (Table 4). In a combined logistic-regression model, copeptin did not statistically significantly improved the area under curve of WFNS score (*P *= 0.125) and modified Fisher score (*P *= 0.164).

**Table 4:** Receiver operating characteristic curve analysis of factors predicting the one-year mortality among 303 patients

	Copeptin	WFNS score	Modified Fisher score
Criterion	>20.5 pmol/L	>3	>3
Area under curve	0.868	0.920	0.927
95% confidence interval	0.824 ~ 0.904	0.884 ~ 0.948	0.892~0.954
Sensitivity	92.9	78.6	88.1
95% confidence interval	80.5 ~98.4	63.2 ~89.7	74.4 ~96.0
Specificity	70.1	90.4	86.6
95% confidence interval	64.2 ~ 75.6	86.2~93.7	81.8~90.5
Odds ratio	29.949	34.613	47.743
95% confidence interval	8.998~99.796	14.876~80.563	17.579~129.667
+ likelihood ratio	3.11	8.20	6.57
95% confidence interval	2.8 ~3.5	7.0~ 9.7	5.8 ~7.4
- likelihood ratio	0.10	0.24	0.14
95% confidence interval	0.03~ 0.3	0.1 ~0.5	0.06 ~0.3
*P *value	Reference	0.197	0.155

WFNS indicates World Federation of Neurological Surgeons.

### In-hospital mortality prediction

Thirty-two patients (10.6%) died from SAH in the hospital. Higher plasma copeptin level was associated with in-hospital mortality, as well as other variables shown in the Table 5. When the above variables found to be significant in the univariate analysis were introduced into the logistic model, a multivariate analysis selected WFNS score, modified Fisher score and plasma copeptin level as the independent predictors for in-hospital mortality of patients (Table 6).

**Table 5:** The factors associated with in-hospital mortality

	Non-survivals (*n *= 32)	Survival (*n *= 271)	*P *value
Sex (male/female)	12/20	119/152	0.489
Age (y)	45.4 ± 12.9	43.7 ± 12.3	0.469
WFNS score on admission	3.9 ± 0.7	2.2 ± 1.1	<0.001
Modified Fisher score on admission	4.3 ± 0.6	2.6 ± 0.8	<0.001
Aneurysmal location			0.599
Posterior communication artery	5 (15.6%)	78 (28.8%)	
Internal carotid artery	6 (18.8%)	37(13.7%)	
Anterior communication artery	8 (25.0%)	58 (21.4%)	
Middle cerebral artery	4 (12.5%)	41 (15.1%)	
Anterior cerebral artery	4 (12.5%)	31 (11.4%)	
Posterior cerebral artery	3 (9.4%)	20 (7.4%)	
Vertebral artery	2 (6.3%)	6 (2.2%)	
Surgery	17 (53.1%)	169 (62.4%)	0.310
Aneurysmal size (mm)	11.9 ± 5.1	6.7 ± 4.5	<0.001
Rebleeding	6 (18.8%)	10 (3.7%)	<0.001
Acute hydrocephalus	19 (59.4%)	71 (26.2%)	<0.001
Intracerebral hemorrhage	15 (46.9%)	24 (8.9%)	<0.001
Intraventricular hemorrhage	31 (96.9%)	41 (15.1%)	<0.001
External ventricular drain	30 (93.8%)	79 (29.2%)	<0.001
Angiographic vasospasm	30 (93.8%)	101 (37.3%)	<0.001
Computed tomography ischemia	16 (50.0%)	34 (12.6%)	<0.001
Admission time (hr)	5.4 ± 4.2	4.6 ± 3.6	0.276
Seizure	7 (21.9%)	37 (13.7%)	0.212
Plasma C-reactive protein level (mg/L)	9.0 ± 3.2	6.9 ± 2.6	<0.001
plasma D-dimer level (mg/L)	2.4 ± 1.1	2.0 ± 1.0	0.045
Plasma copeptin level (pmol/L)	31.8 ± 8.5	19.9 ± 8.2	<0.001

Numerical variables were presented as mean ± standard deviation. Categorical variables were expressed as counts (percentage). Numerical variables were analyzed by Mann-Whitney U-test or unpaired Student *t *test. Categorical variables were analyzed by chi-square test or Fisher exact test. n indicates number of patients; WFNS, World Federation of Neurological Surgeons.

**Table 6:** Multivariate analysis of factors predicting the in-hospital mortality among 303 patients

	Odds ratio	95% confidence interval	*P *value
WFNS score on admission	4.973	1.497~13.137	0.003
Modified Fisher score on admission	5.982	2.469~17.149	0.001
Aneurysmal size (mm)	1.218	0.909~1.423	0.103
Rebleeding	1.483	0.882~5.492	0.163
Acute hydrocephalus	1.097	0.711~4.483	0.242
Intracerebral hemorrhage	1.479	0.703~5.176	0.207
Intraventricular hemorrhage	3.94	0.816~9.407	0.096
External ventricular drain	2.009	0.518~5.36	0.148
Angiographic vasospasm	2.930	0.918~7.998	0.091
Computed tomography ischemia	1.482	0.683~5.004	0.315
Plasma C-reactive protein level (mg/L)	1.455	0.673~2.947	0.482
plasma D-dimer level (mg/L)	0.915	0.587~2.936	0.367
Plasma copeptin level (pmol/L)	2.515	1.399~8.229	0.002

The relation of copeptin to the mortality was assessed in a logistic-regression model. WFNS indicates World Federation of Neurological Surgeons.

A receiver operating characteristic curve showed that plasma copeptin level on admission predicted in-hospital mortality of patients statistically significantly (Figure 3B). The predictive value of the copeptin concentration was similar to those of WFNS score and modified Fisher score (Table 7). In a combined logistic-regression model, copeptin did not statistically significantly improved the area under curve of WFNS score (*P *= 0.148) and modified Fisher score (*P *= 0.135).

**Table 7:** Receiver operating characteristic curve analysis of factors predicting the in-hospital mortality among 303 patients

	Copeptin	WFNS score	Modified Fisher score
Criterion	>28.7 pmol/L	>3	>3
Area under curve	0.868	0.893	0.922
95% confidence interval	0.825 ~0.904	0.853 ~ 0.926	0.886~0.950
Sensitivity	78.1	75.0	90.6
95% confidence interval	60.0 ~90.7	56.6 ~88.5	75.0 ~97.9
Specificity	89.7	87.5	84.1
95% confidence interval	85.4 ~ 93.0	82.9~91.2	79.2~88.3
Odds ratio	29.798	20.911	51.254
95% confidence interval	11.847~74.946	8.698~50.270	14.944~105.792
+ likelihood ratio	7.56	5.98	5.71
95% confidence interval	6.3 ~9.1	4.9~ 7.3	5.1 ~6.5
- likelihood ratio	0.24	0.29	0.11
95% confidence interval	0.1~ 0.5	0.1 ~0.6	0.04 ~0.3
*P *value	Reference	0.613	0.246

WFNS indicates World Federation of Neurological Surgeons.

### Cerebrovasospasm prediction

One hundred and thirty-one (43.2%) suffered from cerebrovasospasm in the hospital. Higher plasma copeptin level was associated with cerebrovasospasm, as well as other variables shown in the Table 8. When the above variables found to be significant in the univariate analysis were introduced into the logistic model, a multivariate analysis selected WFNS score, modified Fisher score and plasma copeptin level as the independent predictors for cerebrovasospasm of patients (Table 9).

**Table 8:** The factors associated with cerebrovasospasm

	Vasospasm (*n *= 131)	Non-vasospasm (*n *= 172)	*P *value
Sex (male/female)	61/70	70/102	0.307
Age (y)	43.8 ± 12.2	43.9 ± 12.5	0.973
WFNS score on admission	3.2 ± 1.1	1.7 ± 0.9	<0.001
Modified Fisher score on admission	3.5 ± 1.0	2.2 ± 0.6	<0.001
Aneurysmal location			0.813
Posterior communication artery	33 (25.2%)	50 (29.1%)	
Internal carotid artery	20 (15.3%)	23(13.4%)	
Anterior communication artery	29 (22.1%)	37 (21.5%)	
Middle cerebral artery	22 (16.8%)	23 (13.4%)	
Anterior cerebral artery	16 (12.2%)	19 (11.0%)	
Posterior cerebral artery	7 (5.3%)	16 (9.3%)	
Vertebral artery	4 (3.1%)	4 (2.3%)	
Surgery	86 (65.5%)	100 (58.1%)	0.183
Aneurysmal size (mm)	9.1 ± 5.7	5.8 ± 3.5	<0.001
Rebleeding	10 (7.6%)	6 (3.5%)	0.110
Acute hydrocephalus	71 (54.2%)	19 (11.0%)	<0.001
Intracerebral hemorrhage	26 (19.9%)	13(7.6%)	0.002
Intraventricular hemorrhage	52 (39.7%)	20 (11.6%)	<0.001
External ventricular drain	90 (68.7%)	19 (11.0%)	<0.001
Admission time (hr)	4.5 ± 3.4	4.9 ± 3.8	0.342
Seizure	17 (13.0%)	27 (15.7%)	0.505
Systolic arterial pressure (mmHg)	134.2 ± 23.6	128.7 ± 21.2	0.033
Diastolic arterial pressure (mmHg)	81.1 ± 15.1	77.5 ± 13.8	0.031
Mean arterial pressure (mmHg)	98.8 ± 16.1	94.6 ± 15.3	0.020
Plasma C-reactive protein level (mg/L)	7.9 ± 3.0	6.6 ± 2.4	<0.001
plasma D-dimer level (mg/L)	2.2 ± 1.1	1.9 ± 0.9	0.011
Plasma copeptin level (pmol/L)	25.0 ± 8.4	18.3 ± 8.3	<0.001

Numerical variables were presented as mean ± standard deviation. Categorical variables were expressed as counts (percentage). Numerical variables were analyzed by Mann-Whitney U-test or unpaired Student *t *test. Categorical variables were analyzed by chi-square test or Fisher exact test. n indicates number of patients; WFNS, World Federation of Neurological Surgeons.

**Table 9:** Multivariate analysis of factors predicting the cerebrovasospasm among 303 patients

	Odds ratio	95% confidence interval	*P *value
WFNS score on admission	3.988	1.248~8.909	0.005
Modified Fisher score on admission	4.692	1.627~15.726	0.001
Aneurysmal size (mm)	1.104	0.998~1.247	0.061
Acute hydrocephalus	1.548	0.932~4.877	0.078
Intracerebral hemorrhage	1.709	0.437~5.174	0.514
Intraventricular hemorrhage	2.472	0.911~6.972	0.060
External ventricular drain	1.615	0.604~6.183	0.176
Systolic arterial pressure (mmHg)	1.012	0.989~1.214	0.612
Diastolic arterial pressure (mmHg)	1.003	0.972~1.044	0.622
Mean arterial pressure (mmHg)	1.005	0.901~1.103	0.583
Plasma C-reactive protein level (mg/L)	0.893	0.713~1.082	0.204
plasma D-dimer level (mg/L)	0.829	0.519~1.327	0.595
Plasma copeptin level (pmol/L)	1.292	1.149~1.605	0.025

The relation of copeptin to the mortality was assessed in a logistic-regression model. WFNS indicates World Federation of Neurological Surgeons.

A receiver operating characteristic curve showed that plasma copeptin level on admission predicted cerebrovasospasm of patients statistically significantly (Figure 3C). The predictive value of the copeptin concentration was lower than those of WFNS score and modified Fisher score (Table 10). In a combined logistic-regression model, copeptin did not statistically significantly improved the area under curve of WFNS score (*P *= 0.206) and modified Fisher score (*P *= 0.288).

**Table 10:** Receiver operating characteristic curve analysis of factors predicting the cerebrovasospasm among 303 patients

	Copeptin	WFNS score	Modified Fisher score
Criterion	>23.4 pmol/L	>2	>2
Area under curve	0.792	0.879	0.874
95% confidence interval	0.742 ~0.836	0.837 ~ 0.913	0.831~0.909
Sensitivity	69.2	80.9	84.7
95% confidence interval	60.8 ~77.4	73.1 ~87.3	77.4 ~90.4
Specificity	84.9	79.7	76.7
95% confidence interval	78.6 ~ 89.9	72.9~85.4	69.4~82.2
Odds ratio	7.783	9.488	7.814
95% confidence interval	4.617~13.121	5.597~16.085	4.663~13.096
+ likelihood ratio	4.60	3.98	3.64
95% confidence interval	4.0 ~5.2	3.6~ 4.4	3.3 ~4.1
- likelihood ratio	0.36	0.24	0.20
95% confidence interval	0.2~ 0.6	0.2 ~0.4	0.1 ~0.3
*P *value	Reference	0.002	0.006

WFNS indicates World Federation of Neurological Surgeons.

### Poor neurologic function prediction

Ninety patients (29.7%) suffered from poor neurologic outcome (GOS 1-3) in one year. Higher plasma copeptin level was associated with one-year poor neurologic outcome, as well as other variables shown in the Table 11. When the above variables found to be significant in the univariate analysis were introduced into the logistic model, a multivariate analysis selected WFNS score, modified Fisher score and plasma copeptin level as the independent predictors for one-year poor neurologic outcome of patients (Table 12).

**Table 11:** The factors associated with one-year function outcome

	GOS 1-3 (*n *= 90)	GOS 4-5 (*n *= 213)	*P *value
Sex (male/female)	42/48	89/124	0.433
Age (y)	44.7 ± 11.3	43.5 ± 12.8	0.422
WFNS score on admission	3.6 ± 0.7	1.8 ± 0.9	<0.001
Modified Fisher score on admission	3.8 ± 0.8	2.3 ± 0.7	<0.001
Aneurysmal location			0.291
Posterior communication artery	24 (26.7%)	59 (27.7%)	
Internal carotid artery	13 (14.4%)	30(14.1%)	
Anterior communication artery	17 (18.9%)	49 (23.0%)	
Middle cerebral artery	14 (15.6%)	31 (14.6%)	
Anterior cerebral artery	7 (7.8%)	28 (13.1%)	
Posterior cerebral artery	11 (12.2%)	12 (5.6%)	
Vertebral artery	4 (4.4%)	4 (1.9%)	
Surgery	54 (60.0%)	132 (62.0%)	0.747
Aneurysmal size (mm)	10.4 ± 5.8	5.9 ± 3.7	<0.001
Rebleeding	10 (11.1%)	6 (2.8%)	0.003
Acute hydrocephalus	47 (52.2%)	43 (20.2%)	<0.001
Intracerebral hemorrhage	20 (22.2%)	19 (8.9%)	0.002
Intraventricular hemorrhage	59(65.6%)	12 (5.6%)	<0.001
External ventricular drain	66 (73.3%)	43 (20.2%)	<0.001
Angiographic vasospasm	71 (78.9%)	60 (28.2%)	<0.001
Computed tomography ischemia	28 (31.1%)	22 (10.3%)	<0.001
Admission time (hr)	4.5 ± 3.5	4.8 ± 3.7	0.577
Seizure	19 (21.1%)	25 (11.7%)	0.085
Plasma C-reactive protein level (mg/L)	8.2 ± 3.2	6.7 ± 2.4	<0.001
plasma D-dimer level (mg/L)	2.4 ± 1.2	1.9 ± 0.9	<0.001
Plasma copeptin level (pmol/L)	29.3 ± 9.0	17.8 ± 6.4	<0.001

Numerical variables were presented as mean ± standard deviation. Categorical variables were expressed as counts (percentage). Numerical variables were analyzed by Mann-Whitney U-test or unpaired Student *t *test. Categorical variables were analyzed by chi-square test or Fisher exact test. n indicates number of patients; WFNS, World Federation of Neurological Surgeons; GOS, Glasgow Outcome Scale.

**Table 12:** Multivariate analysis of factors predicting one-year poor functional outcome among 303 patients

	Odds ratio	95% confidence interval	*P *value
WFNS score on admission	4.930	1.997~13.438	0.005
Modified Fisher score on admission	5.743	2.502~16.307	0.001
Aneurysmal size (mm)	1.129	0.782~1.337	0.137
Rebleeding	1.219	0.841~9.090	0.103
Acute hydrocephalus	1.132	0.730~8.004	0.218
Intracerebral hemorrhage	2.670	0.470~14.003	0.314
Intraventricular hemorrhage	2.404	0.817~12.337	0.087
External ventricular drain	1.316	0.718~4.796	0.316
Angiographic vasospasm	1.174	0.790~3.931	0.113
Computed tomography ischemia	1.422	0.849~3.640	0.139
Plasma C-reactive protein level (mg/L)	1.019	0.827~1.213	0.709
plasma D-dimer level (mg/L)	1.182	0.762~1.899	0.418
Plasma copeptin level (pmol/L)	1.253	1.109~1.504	0.001

The relation of copeptin to the poor functional outcome was assessed in a logistic-regression model. WFNS indicates World Federation of Neurological Surgeons.

A receiver operating characteristic curve showed that plasma copeptin level on admission predicted one-year poor neurologic outcome of patients statistically significantly (Figure 3D). The predictive value of the copeptin concentration was similar to those of WFNS score and modified Fisher score (Table 13). In a combined logistic-regression model, copeptin statistically significantly improved the area under curve of WFNS score (*P *= 0.018) and modified Fisher score (*P *= 0.029).

**Table 13:** Receiver operating characteristic curve analysis of factors predicting one-year poor functional outcome among 303 patients

	Copeptin	WFNS score	Modified Fisher score
Criterion	>23.8 pmol/L	>2	>2
Area under curve	0.860	0.909	0.902
95% confidence interval	0.815~0.897	0.871~0.939	0.863~0.933
Sensitivity	82.2	96.7	95.6
95% confidence interval	72.7~89.5	90.6~99.3	89.0 ~98.7
Specificity	79.8	74.7	69.5
95% confidence interval	73.8~85.0	68.3 ~80.3	62.8~75.6
Odds ratio	18.284	85.228	48.941
95% confidence interval	9.684~34.522	25.912~280.325	17.237~139.015
+ likelihood ratio	4.07	3.81	3.13
95% confidence interval	3.6~4.6	3.5~4.2	2.8 ~3.5
- likelihood ratio	0.22	0.045	0.064
95% confidence interval	0.1~0.4	0.01 ~0.1	0.02 ~0.2
*P *value	Reference	0.109	0.178

WFNS indicates World Federation of Neurological Surgeons.

## Discussion

In this retrospective study, we aimed to investigate the plasma copeptin levels in the SAH patients, and therefore, presence of other prior systemic diseases including uremia, liver cirrhosis, malignancy, chronic heart or lung disease, diabetes mellitus and hypertension, that may be associated with plasma copeptin levels [1-7,17], may become the confounding variables, and finally, were excluded. Furthermore, we demonstrated that plasma copeptin levels on admission in the patients were significantly higher than those in healthy controls; and in patients who had poor functional outcome or died in a year or die in hospital, the copeptin levels on admission were significantly higher compared with levels in survivors or patients with good functional outcome. In multivariate logistic regression models of predictors of death and poor functional outcome that included other confounding variables previously reported [18-20], the copeptin levels on admission were an independent predictor. Need to mention, of two patients with basilar tip aneurysms in this study, one refused to treat and the other had missing of follow up. And therefore, they could be included in this study. In addition, in this study, plasma copeptin level for these patients (turning point: 23.8 pmol/L) was similar to that of intracerebral hemorrhage (turning point: 18 pmol/L) [9] or ischemic stroke (turning point: 16.3 pmol/L) [10] in previous reports. Grade III is typically associated with worse poorer outcome. This result was verified by receiver operating characteristic curve analysis with high area under curve. Recently, various risk factors for poor outcome after SAH have been identified, and include age, WFNS grade, aneurysmal size, vasospasm, clot thickness and so on [17]. In this study, aneurysmal size, vasospasm, computed tomography ischemia and so on were highly associated with poor outcome in univariate analysis, but they were not in multivariate analysis. However, WFNS grade, modified Fisher grade and plasma copeptin level were identified as independent predictors for poor outcome. Generally, in few reports, biochemical markers were assessed. In this study, some biochemical markers were included. However, if larger sample size are obtained, these factors such as vasospasm and computed tomography ischemia, which are probably equally important, will be included multivariate model. And therefore, it is possible that different variables included in these studies and study designs led to these differences. In addition, in previous exploratory analysis, aneurysm coiling was associated with less angiographic vasospasm and delayed ischemic neurological deficit than surgical clipping; furthermore, Dumont et al. still suggested whether this is attributable to differences in baseline risk factors between clipped and coiled patients or a true difference remained to be proven [21]. However, our study did not show better outcomes and less angiographic vasospasm for coiled patients. Thus, final conclusion remains to be verified.

Copeptin is co-synthesized with arginine vasopressin in the hypothalamus and is released into the portal circulation of the neurohypophysis. Arginine vasopressin contributes to the regulation of osmotic and cardiovascular homeostasis [22,23]. In addition, arginine vasopressin activates the hypothalamo - pituitary - adrenal axis through potentiation of corticotrophin - releasing - hormone - induced adrenocorticotropic hormone secretion and thus reflects the individual stress response at a hypothalamic level [24,25]. Copeptin concentrations mirror that of arginine vasopressin [26]. Copeptin is known to have prognostic value in a variety of diseases, as it reflects disease severity and thus the chance of recovery. For example, copeptin levels have prognostic implications in patients with hemorrhagic and septic shock, lower respiratory tract infection and acute heart failure [1-11]. Therefore, it has been hypothesized that the close and reproducible relation of copeptin levels to the degree of activation of the stress axis is the basis of its unique usefulness as a prognostic biomarker [8]. In addition, data from experimental studies imply that vasopressin plays a role in brain edema formation as blocking of vasopressin receptors attenuates brain edema in ischemic and traumatic mice models [27-29]. The relationship between vasopressin levels and brain edema development has also been demonstrated in a clinical study of head injured patients [30]. Hence, the implication of copeptin and brain edema formation in SAH remains hypothetical at the moment.

Copeptin comes from the same precursor as mature arginine vasopressin, which is already well associated with hemodynamic changes and patient outcome. The measurement of mature arginine vasopressin, however, is subject to considerable challenges, and has therefore not reached clinical routine in the context of rapid measurements in the critically ill patients. Here, the stability and longer ex vivo half-life of copeptin is a practical advantage, which makes it easier to determine in the clinical laboratory. In this study, a receiver operating characteristic curve showed that plasma copeptin level on admission predicted poor functional outcome and mortality after 1 year, and in-hospital mortality of patients obviously. The area under curve of the copeptin concentration was similar to those of WFNS score and modified Fisher score for the prediction of these poor outcomes. In a combined logistic-regression model, copeptin improved the area under curve of WFNS score and modified Fisher score for the prediction of poor functional outcome after 1 year, but not for the prediction of mortality after 1 year or in-hospital mortality. Therefore, the determination of copeptin in the plasma of patients on admission provides the ability to distinguish between patients with good and bad outcome.

Cerebrovasospasm is regarded as abnormal and prolonged smooth muscle contraction of cerebral arteries; many substances have been involved in the development of cerebral vasospasm following SAH, but the complex mechanism of this arterial narrowing is not yet fully understood [31,32]. Some authors have reported that arginine vasopressin may play a role in the development of cerebral vasospasm [33,34] and ischemic brain edema [35,36]. Arginine vasopressin causes vasoconstriction in rabbit, feline, goat, rat and human [37-41]. In a model of SAH in rats, Delgado et al. demonstrated a biphasic, angiographically visible vasospasm with maximum acute vasospasm at 10 min after SAH and maximum late vasospasm 2 days later [42]. It was also shown that intracisternal injection of AVP determined acute vasospasm with a time-course similar to that seen in normal rats after SAH [34]. These studies sustain a better understanding of the role of arginine vasopressin in the cerebrovasospasm following SAH. Copeptin concentrations mirror that of arginine vasopressin [26]. Coupled with our observation that plasma copeptin level was an independent predictor for cerebrovasospasm of patients, the implication of copeptin and cerebrovasospasm formation in SAH remains hypothetical at the moment. Grade III is typically associated with the formation of vasospasm. This result was verified by receiver operating characteristic curve analysis with high area under curve. However, significantly lower accuracy for the prediction of cerebrovasospasm was found for plasma copeptin level compared with other clinical grade such as WFNS and modified Fisher grade. Hence, plasma levels of copeptin on admission are not recommended for the prediction of cerebrovasospasm after SAH.

## Conclusions

In this study, plasma copeptin level is a useful, complementary tool to predict functional outcome and mortality after aneurysmal subarachnoid hemorrhage.

## Key messages

• In the patients with aneurysmal subarachnoid hemorrhage, plasma copeptin level was substantially higher than that in healthy controls.

• Plasma copeptin level was an independent predictor of functional outcome and death after aneurysmal subarachnoid hemorrhage

• Copeptin level was a useful, complementary tool to predict functional outcome and mortality after aneurysmal subarachnoid hemorrhage.

## Abbreviations

AUC: areas under the receiver operating characteristics curve; CT: computerized tomography; ELISA: enzyme-linked immunosorbent assay; GOS: Glasgow outcome scale; SAH: subarachnoid hemorrhage; WFNS: World Federation of Neurological Surgeons.

## Competing interests

The authors declare that they have no competing interests.

## Authors' contributions

XDZ and JSC contributed to the design of the study and drafted the manuscript and participated in the laboratory work. JSC, FZ and QCL enrolled the patients. GC and JMZ contributed to data analysis and interpretation of the results. All authors read and approved the final manuscript.
